# Does serum progesterone level impact the ongoing pregnancy rate in frozen embryo transfer under artificial preparation with vaginal progesterone? Study protocol for a randomized controlled trial

**DOI:** 10.1186/s13063-021-05953-8

**Published:** 2022-01-03

**Authors:** Lin Haiyan, Yang Gang, Li Yu, Li Lin, Chen Xiaoli, Zhang Qingxue

**Affiliations:** Reproductive Center of Sun Yat-sen Memorial Hospital, Sun Yat-sen University, Guangzhou, China

**Keywords:** Vaginal progesterone, HRT-FET, Ongoing pregnancy rate

## Abstract

**Background:**

In previous retrospective studies, low serum progesterone level on the embryo transfer day is associated with lower clinical pregnancy and ongoing pregnancy rates. Whether adding progesterone in low serum progesterone patients can rescue the outcome, there is no sufficient evidence from randomized controlled studies.

**Methods:**

This trial is a clinical randomized controlled study (high serum progesterone vs low serum progesterone 1:1, 1:1 randomization ratio of intervention vs the control group with low serum progesterone). The eligible hormone replacement therapy—frozen embryo transfer (HRT-FET) cycles, will be recruited and randomly assigned to two parallel groups when serum progesterone is < 7.24μg/l on the day of embryo transfer for D3. The intervention group will be extrally given intramuscular progesterone 40 mg per day from D3 to 8 weeks of gestation if clinical pregnancy. The primary outcome is the ongoing pregnancy (beyond 12 weeks of gestation) rate.

**Discussion:**

The findings of this study will provide strong evidence for whether the progesterone addition from the D3 in low serum progesterone patients can improve the outcome in the HRT-FET cycle.

**Trial registration:**

ClinicalTrials.govNCT04248309. Registered on January 28, 2020

## Background

Nowadays, frozen embryo transfers(FET) becomes more and more popular in assisted reproductive technology (ART) because it can reduce the ovarian hyperstimulation syndrome (OHSS) rate in fresh cycles especially when agonist trigger in antagonist cycles and elective freezing of all embryos are used. Many centers prefer hormone replacement therapy (HRT)-FET because it can be flexible on which day the exogenous progesterone (P) is added very precisely and make embryo transfer (ET) day to be scheduled. It is essential for favoring synchrony between the embryo and the endometrium during the window of implantation. In HRT-FET using exogenous P is necessary, as a functioning corpus luteum which can produce endogenous P is not present in programed cycles. In the preimplantation period, progesterone plays an important role with 17b estradiol (E) to make the endometrium competent for embryo implantation.

HRT-FET begins with exogenous estrogens from day 2 or 3 of the patients’ menstruation to imitate the endometrium proliferative phase. Once the endometrium thickness by vaginal ultrasound reaches about 7 to 8 mm, exogenous P is supplemented to transform the endometrium to the secretory phase. The starting day of P is so-called day 0 for embryo transfer. Up to now, there is continuing debate about optimal serum estradiol and P levels in HRT-FET cycles. However, the optimal serum P level associated with the best cycle outcome is required to be established. For this reason, recently, few retrospective studies were published and concluded that serum P levels on the day of ET or the day of pregnancy test were related with ongoing pregnancy rate (OPR) [1–3]. Previous retrospective study also showed an increase in P dose after ET was insufficient to rescue pregnancy rates even in the overweight and obese patients [4].

There are several different kinds of exogenous progesterone such as intramuscular, orally, vaginally, and trans-rectum [5]. The vaginal progesterone has been most widely used in ART because of its convenience and uterine first-pass effect. Vaginal P rapidly reaches the systemic circulation, and the steady state in the serum is achieved around 48–50 h such as Utrogestan 600 mg in a day, so its serum measurement on the day of ET seems reliable. Besides, a previous study showed that increasing doses of vaginal P do not proportionally increase serum concentrations [6]. In fact, the uptake, absorption, and metabolism of progesterone can vary among individualized patients [3]. If there is an optimal serum progesterone level, this problem might be solved by increasing the P dose from the very beginning or other optimal times, but has not yet to be demonstrated. The latest prospective study concluded that low serum progesterone on the day of embryo transfer was associated with a diminished ongoing pregnancy rate in oocyte donation cycles after artificial endometrial preparation [7]. We raised the question that if progesterone added on the day of ET can improve the outcome. So, it is necessary to explore this issue in the prospective controlled study.

According to this background, we design a prospective study to explore if serum P levels on the day of ET are related with clinical pregnancy and ongoing pregnancy rate, and additional progesterone intervention is helpful to improve the outcome.

## Methods/design

### Study design

**Fig. 1:**
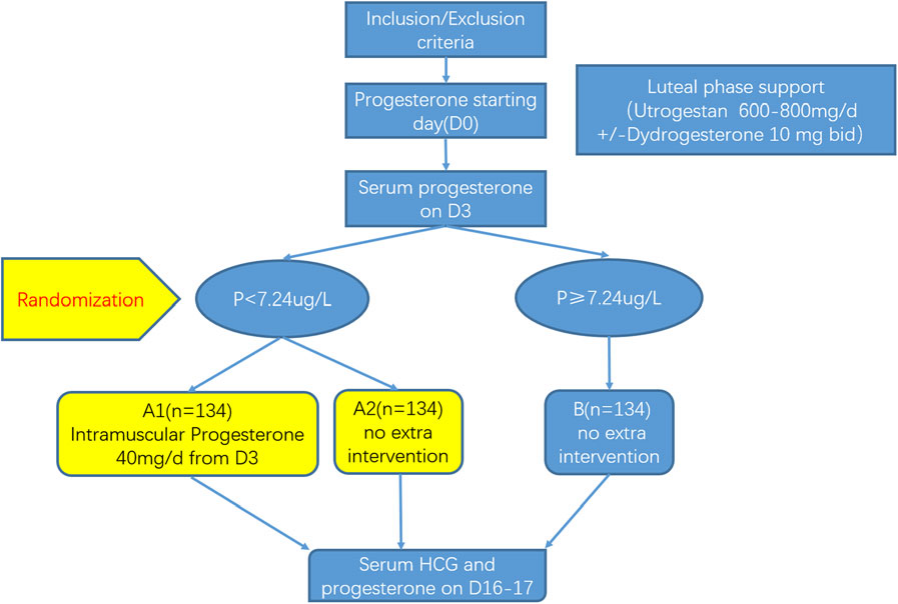
Flowchart of the study design

The study is designed as a randomized controlled trial. Eligible patients will be recruited in this study and checked the serum progesterone on the day of embryo transfer. According to the serum progesterone, the patients with low serum progesterone will be randomly assigned to the intervention group or the control group with a 1:1 ratio (see Fig. 1).

### Study sites

The prospective randomized controlled study will be performed at the Reproductive Center, Sun Yat-sen Memorial Hospital of Sun Yat-sen University. It is estimated that there will be 432 HRT-FET cycles recruited to compare the ongoing pregnancy rate between the intervention group and the control group.

The Good Clinical Practice guidelines and the Declaration of Helsinki will be followed by the study. The study has been approved by the ethics committees of Sun Yat-sen Memorial Hospital of Sun Yat-sen University (2019 IRB approval no. 5).

The investigators will screen the HRT-FET patients on the day of decision with embryo transfer day. If the patients are voluntary to participate and eligible with inclusion/exclusion criteria, informed consent will be obtained from each patient before the serum progesterone test.

Reporting of the study results will follow the 2010 revised Consolidated Standards of Reporting Trials (CONSORT) statement [8].

**Fig. 2:**
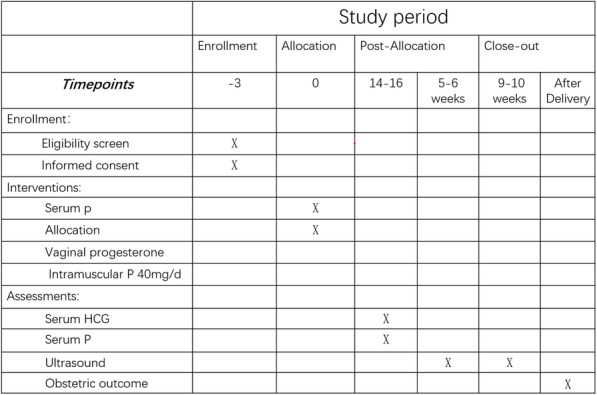
Standard Protocol Items: Recommendations for Interventional Trials (SPIRIT) figure

All the main measurements and critical time points of data collection can be found in the Standard Protocol Items: Recommendations for Interventional Trials (SPIRIT) (Fig. 2).

### Training of investigators

All investigators have achieved the certifications of Good Clinical Practice before the study starts. The pre-trial training of the study protocol will be conducted for all the participant investigators.

### Study population

This study will recruit the patients who are planned as HRT-FET including GnRHa downregulated plus HRT-FET cycles.

### Patient population

This study will recruit infertile patients undergoing HRT-FET cycle. All the patients will be performed a single or double embryos transfer in the cleavage or blastocyst stage. The eligibility criteria are as follows.

### Inclusion criteria

The following are the inclusion criteria:

1. HRT-FET cycles, including GnRHa-HRT-FET.
2. Age < 41 years old.
3. BMI < 30 kg/m^2^.
4. TSH and PRL normal (0.27–4.0 mu/L for TSH and < 26 ng/mL for PRL)
5. Endometrium thickness ≥ 8 mm on D0.
6. Embryo transfer completed with at least 1 top-quality embryo (7–9 cell grade 1 or D5 beyond grade 3) within 2 embryos.
7. Luteal phase support: transvaginal progesterone (Utrogestan) 600–800 mg/day with or without oral progesterone (dydrogesterone) 10 mg bid.
8. Each patient is recruited only once.

### Exclusion criteria

The following are the exclusion criteria:

1. History of moderate and severe uterine adhesion
2. Presence of hydrosapinx diameter > 2 cm
3. Endometriosis at stages III–IV
4. Recurrent implantation failure (> 3 times of embryo transfer cycle)
5. PGT cycles

### Withdrawal criteria

Participation in the study is totally voluntary. The subjects can withdraw from the study at any time, and they will still receive standard medical care.

### Sample size calculation

A power analysis was performed according to the results from our own retrospective analysis, concluding a significant decrease in clinical pregnancy rate (CPR) when progesterone was < 7.24 μg/L (the first quartile) of 34.67% compared with ≥ 7.24 μg/L of 51.55% (*P* = 0.029).

The sample size was calculated in a two-sided test (80% statistical power, 95% confidence interval).

Low serum progesterone group: 1:1 intervention (A1) and control group (A2); high serum progesterone group: 1:1 to control group (B)

Group sample sizes are separated: 134 in group A1 and 134 in group A2 and 134 in group B. The test statistic used was the two-sided Fisher’s exact test. The significance level of the test was targeted at 0.05.

The formula is as follows:

$$ n=\frac{{\left({Z}_{1-\alpha }+{Z}_{1-\beta}\right)}^2\times \left({p}_1\left(1-{p}_1\right)+{p}_2\left(1-{p}_2\right)\right)}{{\left(\varepsilon -\delta \right)}^2} $$

After considering 5% of dropouts, 432 patients were recruited in total.

Age, BMI, endometrial thickness, serum E2, and number of transferred embryos were included in the analysis, regardless of their *P* values. Biochemical pregnancy is defined as serum HCG level ≥ 20 U/L. Clinical pregnancy is defined as observable gestation sac by transvaginal ultrasound. Ongoing pregnancy is defined as clinical pregnancy beyond gestational week 12.

### Randomization and blinding

Randomization is carried out in concealed envelopes by a computer-generated randomization list generated by a research assistant who is not involved in the participant’s clinical management. Randomization will be done in a 1:1 ratio in blocks, stratified according to D3 or D5 embryo transfer. On the D3 of the HRT-FET cycle, the investigators get a concealed envelop by different blocks in sequence to obtain the randomization numbers and allocate groups for the eligible patients. Participants will be randomized into one of the two groups on the D3 when serum progesterone is less than 7.24μg/L, if the intervention group has intramuscular progesterone 40 mg per day from D3 to weeks 8 if clinical pregnancy, and if the control group has no extra intervention. The clinicians, nurses, and the participants all will not be blinded to the treatment allocation. The statistician is blinded to the group allocation.

### Procedures

#### Study protocol (endometrial preparation)

All the included patients receive HRT for endometrial preparation. Treatment starts on day 2 or day 3 of menstruation with oral estradiol valerate administration (Progynova®, Bayer Guangzhou, China)—constant dose 4–8 mg/d or step-up increasing dose protocols, and the endometrium thickness will either be checked once during the first 5–7 days. The estradiol dose will be adjusted accordingly. After about 10–14 days of estrogens, a transvaginal ultrasound will be performed to measure the endometrial thickness and to confirm a triple layer pattern. Patients are considered ready for ET once endometrial thickness reaches 8 mm, and quiescent ovaries are detected by ultrasound, serum E2 > 150 pg/mL, and the serum P level < 2 ng/mL. Transvaginal P (Utrogestan® Besins, France) will be administered 3 or 5 days before embryo transfer at a dose of 200 mg tid or 400 mg bid Utrogestan vaginally. Transvaginal progesterone will be started 3 days before embryo transfer for patients who will have day 3 embryo transferred or 5 days before embryo transfer for those who will have day 5 embryo transferred. Serum progesterone will be tested on the morning of D3 (after 3 days of progesterone addition) after the first dose of progesterone, approximately 2 h beforehand. In case of pregnancy, HRT was maintained until gestational weeks 10. The estrogen would be gradually decreased with dosage after gestation at 8 weeks. The progesterone dosage would be not changed.

#### Selecting patients

On the first day of progesterone supplementation (day 0), eligible patients are selected for this study. After checking all the inclusion and exclusion criteria, patients will be informed about the nature of the study, read the informed consent (IC) form, and decide if they wish to participate. After signing the IC form, a blood test of progesterone is performed on day 3. According to the serum progesterone level on day 3, if P is ≥ 7.24 ng/mL, continue with the transvaginal progesterone until the pregnancy test on days 14–16. If P < 7.24 ng/mL, according to the randomization numbers, the patient is randomized to either non-intervention control group or intervention group with intramuscular progesterone 40 mg until the pregnancy test on days 16–17. This protocol will continue until gestation week 8 and withdraw the intramuscular progesterone.

The included patient should test the serum progesterone level on D3 no matter which day the embryo transfer will be performed. According to the progesterone level, there are two groups:

1. Group A: serum progesterone < 7.24μg/L, followed by randomized:
  - A1: plus additional treatment (intramuscular progesterone 40 mg per day from D3)
  - A2: without additional treatment
2. Group B: serum progesterone ≥ 7.24μg/L, without additional treatment

Samples will be tested by the Beckman chemiluminescence method (Beckman DXI800, Roche, USA). The intra- and inter-assay variation coefficients for the P determinations were 6.11–11.19% and 7.51–9.57%, respectively, for *P* values between 1.26 and 22.61 ng/mL. The intra- and inter-assay variation coefficients for the E2 determinations were 2.4–10.5% and 2.4–9%, respectively, with a measurement range of 50–120 pg/mL.

#### Endpoints

All the parameters were defined according to the ICMART and WHO Revised Glossary on ART [9].

The primary endpoint was OPR beyond gestational weeks 12 in HRT-FET cycles.

The secondary endpoints were serum P (ng/mL) and estradiol (E2, pg/mL) on D0, endometrial thickness (mm), implantation rate (number of gestational sacs seen in a transvaginal ultrasound/number of embryos transferred per patient), clinical pregnancy (presence of at least one gestational sac intrauterine in ultrasound), and miscarriage rate (any clinical pregnancy lost before gestation weeks 12).

- Pregnancy: positive pregnancy test, HCG level beyond 20 U/L after 14 days of ET

- Clinical pregnancy: the presence of intrauterine gestational sac on pelvic ultrasound at 6 weeks of gestation

- Biochemical pregnancy: a pregnancy diagnosed only by the detection of HCG in serum or urine and that does not develop into a clinical pregnancy

- Implantation rate: number of intrauterine gestational sacs per embryo transferred

- Ongoing pregnancy: viable pregnancy beyond gestation 12 weeks

- Multiple pregnancy, ectopic pregnancy, miscarriage, pelvic infection, fetal or congenital defects, obstetric complications, and birth weight of babies

#### Data collection and follow-up protocol

All data will be recorded in CRF and will be collected at four specific moments: upon recruitment (V0), on the D3 randomization (V1), after the ultrasound at 6–7 weeks’ gestation to confirm the clinical pregnancy (V2), and after a telephone call beyond 12 weeks of gestation and until delivery for follow-up of women who have a clinical pregnancy confirmed at 6–7 weeks (V3).

#### Assessment of safety

We do not expect to have undesirable or serious undesirable events related to the study and its proceedings. Indeed, all procedures included in this study are standard procedures and will be performed in accordance with the national guidelines and recommendations. HRT-FET cycle is common in clinical practice. Adverse effects such as local tenderness, swelling, nodular formation, and even local tissue necrosis are few with the intervention group after intramuscular progesterone injection. These adverse effects will be recorded.

### Statistical analysis

Statistical analysis will be performed by using the Statistical Package for the Social Sciences version 22.0 (SPSS Inc., Chicago, IL, USA). A descriptive analysis of the population’s characteristics will be performed. Demographic features of the two study groups will be compared. The chi-square test and Fisher’s exact test will be used for categorical variables. ANOVA test or the Kruskal-Wallis *H* test will be used to compare the continuous variables between the two groups. Comparison of categorical variables will be expressed as numbers with percentages. The results of continuous variables will be reported as mean values and standard deviations, or medians with 25th and 75th percentile. All statistical tests will be bilateral, and a *P* value < 0.05 will be considered statistically significant.

### Expected repercussions

If OPRs in the intervention group with low serum progesterone are significantly higher than that in the control group, it will provide a valuable evidence for HRT-FET practice. If OPRs were to be comparable between the three groups, the serum progesterone level is not predictive of the outcome.

## Discussion

To date, this is the first RCT to compare the OPR between the intervention and control groups with low serum progesterone in HRT-FET cycles. No strong evidence can be found on this issue.

HRT-FET is popular all over the world. Although there is no sufficient evidence that shows which endometrium preparation protocol is the best, the higher miscarriage rate in HRT-FET cycles attracts researchers’ attention [3, 5, 7, 10–12]. Lately, several retrospective analyses revealed that there was a cutoff of serum progesterone for the clinical outcomes [1, 2, 7, 13, 14].

However, there is currently a lack of guidelines on the use of HRT-FET, such as estrogen regimens, progesterone dosages, duration, and different routes. At present, vaginal progesterone is the most widely used in the world. Due to the first-pass effect of the uterus, vaginal administration can make the concentration of progesterone in the uterus higher. In recent years, the literatures have pointed out the importance of blood progesterone measurement to determine whether there is a deficiency of progesterone. Regardless of the method of administration, it seems that a large proportion of patients do not reach the sufficient progesterone concentration required for successful implantation and sustained pregnancy. Of course, the timing of progesterone testing and the ideal blood progesterone concentration have not yet been determined, and the treatment strategy based on the serum progesterone is still under study. More and more researchers have proposed that individualized progesterone dosage and route of administration can reduce miscarriage and improve prognosis.

In the HRT-FET cycle of egg donation, micronized progesterone (Utrogestan 400 mg/12 h) is administered vaginally [13], and the progesterone on the 5th day of vaginal progesterone administration is less than 35 nmol/L (< 11 ng/mL). Patients had a significantly lower ongoing pregnancy rate (OPR) compared with progesterone exceeding 35 nmol/L (> 11 ng/mL) (35.3% vs 55.6%, RR 0.64; *P* = 0.005). Alsbjerg et al. [15] obtained the same cutoff value. In a cohort study, 244 HRT-FET cycles used 90 mg vaginal progesterone gel (Crinone) three times a day for luteal phase support. OPR in the group with < 35 nmol/L progesterone (38%) was significantly lower than > 35 nmol/L progesterone group (51%) (*P* = 0.043). In addition, in the logistic regression analysis, adjusted with the woman’s age, BMI, smoking, number of embryos transferred, and days of blastocysts (day 5 or 6), the OPR of the low progesterone group (< 35 nmol/L) significantly reduced (OR 0.54, 95% CI 0.32–0.91; *P* = 0.022), the ongoing pregnancy rate was reduced by 14% (95% CI − 26~− 2%; *P* = 0.024). It is reported that 50% of patients have low progesterone levels (10.6 ng/mL; 33.8 nmol/l) [16]. After using vaginal progesterone, it is suggested that insufficient progesterone supplementation during the HRT-FET cycle is an important clinical problem. The latest prospective study with the largest sample size included 1155 HRT-FET cycles using micronized progesterone. The results suggest that the optimal progesterone for ongoing pregnancy is 28 nmol/L (8.8 ng/mL) [17].

However, on the third day after the conversion of progesterone, it is found that the progesterone is lowered. What should be done next, cancel the transfer cycle, continue the transfer with progesterone as originally planned, or delay the transfer time are issues worthy of clinical research. Our study is based on the results of a single-center retrospective data analysis, combined with the progesterone results on the pregnancy test day and the clinical pregnancy outcome analysis. As the cutoff for optimal serum progesterone is inconsistent in various reports. It may be related to the characteristics of patients and differences in laboratory tests of progesterone. Our own retrospective analysis following quartiles are used as cutoff values to estimate the sample size and design a randomized controlled clinical study. The 3-day low cutoff progesterone patients were randomly divided into groups to receive intramuscular progesterone 40 mg, whether to improve the clinical outcome, hoping to provide a basis for clinical decision-making.

## Trial status

Ethical committee of Sun Yat-sen Memorial Hospital approval has already been obtained (2019 IRB approval no.5). The www.clinicaltrials.gov registration number is NCT04248309. The study was conceived and designed in May 2019. Enrollment began in April 2020 and was expected to end in August 2021. At the time of manuscript preparation, more than 300 subjects had been enrolled. Enrollment in this study was ongoing at the time of manuscript submission.

## Data Availability

The datasets generated during the current study are available from the corresponding author on reasonable request.
